# Deflection and Performance Analysis of Shape Memory Alloy-Driven Fiber–Elastomer Composites with Anisotropic Structure

**DOI:** 10.3390/ma17194855

**Published:** 2024-10-02

**Authors:** Anett Endesfelder, Achyuth Ram Annadata, Aline Iobana Acevedo-Velazquez, Markus Koenigsdorff, Gerald Gerlach, Klaus Röbenack, Chokri Cherif, Martina Zimmermann

**Affiliations:** 1 Institute of Materials Science, Faculty of Mechanical Science and Engineering, TUD Dresden University of Technology, Helmholtzstraße 7, 01069 Dresden, Germany; martina.zimmermann@tu-dresden.de; 2 Fraunhofer Institute of Material and Beam Technology IWS, 01277 Dresden, Germany; 3 Institute of Textile Machinery and High Performance Material Technology, Faculty of Mechanical Science and Engineering, TUD Dresden University of Technology, 01062 Dresden, Germany; achyuth_ram.annadata@tu-dresden.de (A.R.A.); chokri.cherif@tu-dresden.de (C.C.); 4 Institute of Control Theory, TUD Dresden University of Technology, 01062 Dresden, Germany; aline_iobana.acevedo_velazquez@tu-dresden.de (A.I.A.-V.); klaus.roebenack@tu-dresden.de (K.R.); 5 Institute of Solid-State Electronics, Faculty of Electrical and Computer Engineering, TUD Dresden University of Technology, Mommsenstraße 15, 01069 Dresden, Germany; markus.koenigsdorff@tu-dresden.de (M.K.); gerald.gerlach@tu-dresden.de (G.G.)

**Keywords:** anisotropy, digital image correlation, elastomer, shape memory alloy

## Abstract

Due to their advantageous characteristics, shape memory alloys (SMAs) are prominent representatives in smart materials. They can be used in application fields such as soft robotics, biomimetics, and medicine. Within this work, a fiber–elastomer composite with integrated SMA wire is developed and investigated. Bending and torsion occur when the SMA is activated because of the anisotropic structure of the textile. The metrological challenge in characterizing actuators that perform both bending and torsion lies in the large active deformation of the composite and the associated difficulties in fully imaging and analyzing this with optical measurement methods. In this work, a multi-sensor camera system with up to four pairs of cameras connected in parallel is used. The structure to be characterized is recorded from all sides to evaluate the movement in three-dimensional space. The energy input and the time required for an even deflection of the actuator are investigated experimentally. Here, the activation parameters for the practical energy input required for long life with good deflection, i.e., good efficiency, were analyzed. Different parameters and times are considered to minimize the energy input and, thus, to prevent possible overheating and damage to the wire. Thermography is used to evaluate the heating of the SMA wire at different actuation times over a defined time.

## 1. Introduction

Elastomer composite structures are a promising basis for adaptive structures and soft robots [1]. Due to the high reversible elongation of the elastomer matrix, they allow a high degree of flexibility for the shaping of different forms with complex and high deformations [2,3]. Depending on specific requirements, multiple ways exist to actuate the structures and generate corresponding forces. In particular, electroactive polymers [4,5], pneumatic structures [6], and shape memory alloys (SMAs) [7,8] are employed. SMAs are predestined to be used in smart polymer composites because of their unique properties. They have controllable reactivity, high energy density, and low power consumption [9]. SMA applications are frequently realized with SMA wires, which can be incorporated in a space-saving manner. They are easily available due to high-demand production [10]. Nickel–titanium (Ni-Ti) alloys are particularly important SMA materials due to their high actuation strain, high force generation, good corrosion resistance, and better biocompatibility properties compared to other SMA materials [11]. These properties make them suitable for applications in medical technology, where they are, e.g., employed in minimally invasive surgical instruments and stents [12,13]. Moreover, there are numerous developments in soft robotics, such as flexible gripper systems, like those developed by Do et al. [14] and Rodrigue et al. [15], which are undergoing continuous improvements.

SMAs are activated thermally via an external heat source, such as a heating element, or electrically through Joule heating via a voltage source. Electrical excitation is the most employed methodology for industrial applications, including medical devices and industrial actuators. When the power supply is connected, the current flows through the alloy, resulting in heating due to its electrical resistance [16]. As the temperature changes, so does the resistance. A phase transition from martensite to austenite occurs as soon as the temperature reaches the transition point (austenite temperature). This results in a corresponding change in resistance and deformation due to the phase change. Upon the deactivation of the electrical signal, the wire cools and the resistance undergoes a reverse change as the wire transitions back to the martensite phase [17].

There is a distinction between the one-way and two-way shape memory effects. This work describes the one-way shape memory effect, whereby the activation and subsequent heating of the SMA alloy results in a deformation. Once the electrical signal has been terminated and the wire cools down, it returns to its original configuration.

Another benefit of electrical activation is a localized limitation of the heating of the whole structure due to the direct warming of the alloy. This avoids additional stress on the remaining components of the overall system. Furthermore, integration into an electronic system is facilitated, thereby enabling the implementation of control strategies with corresponding feedback [18]. Various approaches can be used for different control applications. One method is to record the resistance, which allows the monitoring of functionality, as the temperature change is recorded indirectly [19]. Another approach is to track the position and, hence, to determine the subsequent limitation of the structural movement. Such an array was presented by Asheafiuon et al. to control the movement and avoid the overshooting of a three-link SMA alloy-actuated robot [20].

Although activation by a power supply can be more precisely controlled than by a thermal energy source, it presents several challenges. These include controlling the exact heating rate, avoiding overheating, and maintaining a constant output over repeated cycles. Overcoming these challenges is critical to ensure the actuator’s reliable operation and to prevent potential damage to the SMA material.

Several promising material combinations and manufacturing approaches have been identified to fabricate suitable structures for producing, constructing, and designing flexible, intelligent actuators. In addition to the drive mechanism, the actuator design and manufacturing process play an important role in the actuator’s ability to deform. Manufacturing processes, like 3D printing for flexible, rapid prototyping [21,22] and the production of bone-like structures, like that of Song et al. for a swimming soft robotic turtle [23], are in high demand.

Another approach to manufacturing intelligent, innovative structures that respond flexibly to their environment is 4D printing. The main challenges are the increased complexity of production and the careful selection and combination of materials. This form of additive manufacturing enables the creation of structures that dynamically react to environmental stimuli, such as temperature, light, or humidity [24,25].

The utilization of real-time studies and live monitoring is becoming increasingly prevalent in the monitoring of increasingly complex manufacturing processes. This enables the regulation of processes in real time or the gaining of deeper insight into mechanisms that have yet to be fully characterized. Camera and laser techniques are particularly well suited to this purpose. For example, Priyadarshi et al. employed optical high-speed and ultrafast X-ray imaging to investigate the ultrasonic atomization of water droplets on a vibrating plate and a horn surface [26]. Similarly, Rurup and Secor presented a method for the real-time monitoring of light scattering using an optic cell for quality control in aerosol jet printing [27].

A particularly suitable composite for a soft, flat actuator structure is an elastomer matrix with textile reinforcement. For instance, Lohse et al. [28] described and simulated an actuator demonstrating active bending deformation constructed from glass fiber-reinforced knitted fabric infiltrated with liquid silicone, serving as a bending beam. Due to the infiltration process, this structure can be produced with a low profile. A similar approach can be employed to achieve torsional bending in flat structures by varying the reinforcement layers and fiber orientations [10].

This paper presents the results of the characterization of a fiber-reinforced soft actuator driven by SMA wire, for which this manufacturing approach was used. In preliminary tests, the results obtained were compared with simulations of the actuator deformation using ANSYS [29].

In the current work, an experimental analysis, optimizing the electrical activation parameters to balance out activation energy and actuator deflection, is presented. It addresses these challenges by investigating suitable activation settings to contribute to the development of more robust and, therefore, more durable actuator systems. For the deflection analysis a combined test stand consisting of a multi-sensor camera system with four camera pairs and an IR camera is used. This allows the study of the combined bending and torsional movements of the actuator via digital image correlation (DIC) while simultaneously monitoring the temperature of the wire, thereby enabling a more precise investigation of the behavior of the SMA wire.

## 2. Materials and Methods

### 2.1. Actuator Design and Manufacturing

**Figure 1:**
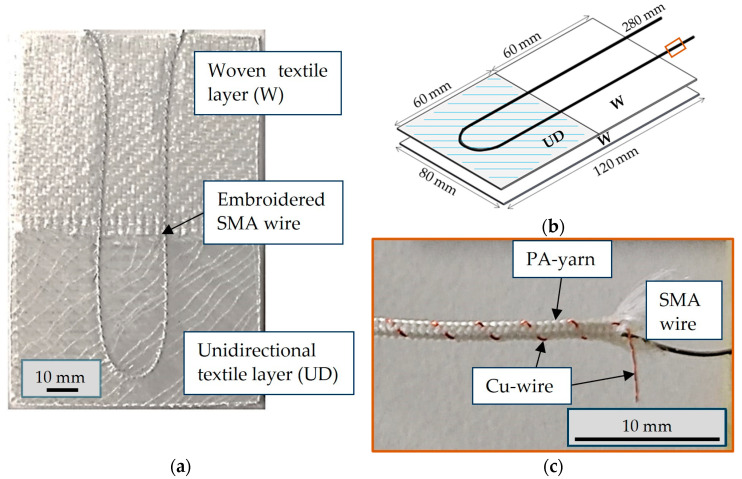
SMA fiber elastomer actuator: (**a**) layout and structure; (**b**) dimensions of the specimen; (**c**) braided Ni-Ti SMA core–sheath structure with copper wire and PA yarns.

The structure and dimensions of the analyzed actuator under investigation are presented in Figure 1.

The manufacturing process of the samples consisted of the following steps:

1. Overbraid the SMA wire with polyamide yarn (PA yarn) and copper wire (cu wire);
2. Place the glass fiber textile layers;
3. Tailor the fiber placement of the braided SMA wire on the textile;
4. Infiltrate the textile with polydimethylsiloxane (PDMS) to form the soft actuator with vacuum-assisted infusion process (VARI process);
5. Cut the sample to size.

The SMA was a nitinol alloy wire with a diameter of 0.3 mm (SmartFlex^®^ 300 µm) from SAES Getters (Milan, Italy). The wire consists of 54.8 m% nickel (Ni) and small amounts of copper (Cu), iron (Fe), niobium (Nb), and cobalt (Co). The SMA wire is drawn cold and then annealed. The manufacturer supplied the SMA wire in a pre-stressed state, which is suitable for actuators when subjected to thermal activation.

Lohse et al. have shown that the SMA wire exhibits a pre-strain of around 4%, with austenite start (A_s_) and final (A_f_) temperatures of 78 °C and 98 °C, as well as the martensite start (M_s)_ and end (M_f_) temperatures of 82 °C and 52 °C [28].

A braid of polyamide and copper wires is made around the Ni-Ti SMA wire using an RU 2/12–80 braiding machine (August Herzog Maschinenfabrik GmbH & Co. KG, Osnabrück, Germany). Due to the braiding, there is no direct contact between the wire and the surrounding materials, and the SMA is not restricted in its ability to contract and expand.

The textile matrix consists of two layers, as shown in Figure 1b. The primary layer of the glass fiber textile matrix was constructed using a 2 × 2 twill fabric comprising 272 tex fibers and was 0.18 mm thick (VR35, Lange + Ritter GmbH, Krefeld, Germany). The second layer was divided into two sections. The upper section comprised the same textile used for the primary layer, while the lower section consisted of a diagonally stitched unidirectional fiber layer comprising 1200° tex fibers. The lower, unidirectional part determines the deflection, while the woven part is responsible for adjusting the partial stiffness of the actuator. The textile layers were placed, and the braided wire was stitched onto the textile base in a central U shape using tailored fiber placement with 27 dtex polyester binder yarn.

The two-component liquid silicone polydimethylsiloxane Sylgard 184™ serves as the elastomer matrix component of the composite material. Silicone was degassed and poured onto the specimen before infusion to prevent air bubble formation. The composite structure was produced using the VARI process. The siliconized samples were cured for 48 h at room temperature.

### 2.2. Activation of the Structure

**Figure 2:**
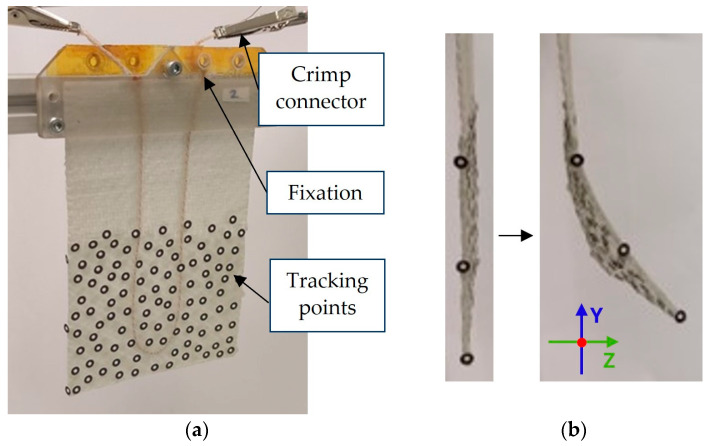
Electrically connected actuator: (**a**) front side with tracking point pattern; (**b**) side view of the actuator in initial and deflected state.

As mentioned in Section 1, the actuator is deformed by heating the SMA wire. This is caused by the intrinsic change in electrical resistance triggered by electrical activation. The power supply’s output wires were attached to the SMA wire with crimp connectors (Figure 2). The sample and SMA wire were clamped to a fixation clamp.

No external force was exerted to investigate the influence of the actuation. A laboratory power supply unit (BASETech, BT-305, Hirschau, Germany) with adjustable voltage and adjustable current limitation was employed for the activation. A Simulink program was used for the cyclic control of the actuator, which controlled an Arduino microcontroller connected to the power supply. Different current limits between 1.5 A and 3.0 A and a voltage between 6 V and 10 V were tested to evaluate the actuator’s changing deformation.

The experimental procedure comprised the following steps:

1. Load cycling at the beginning of the test to stabilize the actuator behavior;
2. Carrying out of preliminary tests based on previous investigations [30] and carrying out of subsequent adaptations;
3. Selection of parameters:
  - Switch-on time of 5 s and switch-off time of 45 s as main setting;
  - Doubling of the switch-on and switch-off times for two electrical settings: 10 s switch-on time and 90 s switch-off time.

### 2.3. Measurement Methodology

**Figure 3:**
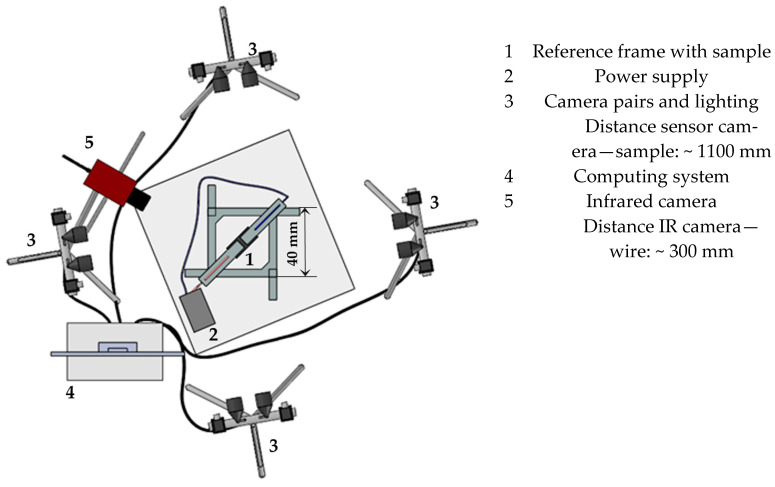
Top view of the test setup with multi-sensor camera system and IR camera for temperature measurement.

The deformation behavior of the soft actuators was analyzed by means of a multi-sensor camera system with four camera pairs (Aramis 5M, Carl Zeiss GOM Metrology GmbH, Braunschweig, Germany). This system enables the simultaneous measurement of different perspectives with different measuring fields. The system’s main application is to analyze images recorded concurrently within a unified coordinate system [31]. Due to the significant deflection of the actuators, varying numbers of camera pairs are required for the evaluation, depending on the scenario. The deformation was tracked with reference points distributed on the test specimen surface. These points are found again in the individual views of the camera pairs, i.e., if they are lost in the field of view of one camera pair but appear in another, a mapping is carried out. This enables the monitoring of substantial deformations, which would not be trackable with a single static camera pair. Another possibility is the simultaneous observation of distinct regions within a single sample. The schematic measurement setup is shown in Figure 3. To ascertain the extent of actuator movement, reference points were distributed randomly across the surface in the area to be measured (Figure 2a). After camera calibration using a calibration plate, the motion of the actuator was recorded at a resolution of 2448 × 2048 pixels with a recording rate of 2 Hz per sensor camera pair. The recording was initiated by one camera, which triggered the simultaneous start of the other ones. The detection field was constrained by the reference frame, which had a measuring volume of 40 × 40 mm.

The actual voltage U and current I achieved during activation were recorded with a multimeter (Keithley DAQ6500-7700, Keithley Instruments, Cleveland, OH, USA) to validate the activation signals. The signals were measured at the crimp connector to be compared to the conditions set at the power supply. Due to losses such as contact resistances at the clamps, these may deviate from the values set at the voltage source. Data from the activation cycles and an extended switch-on for 15 s to control the change over a longer time were recorded.

The recorded data were used to calculate the resistance *R* of the SMA wire according to Ohm’s law.

(1) $$R=\frac{U}{I}$$

A four-point measurement with the same setting was used to control the resistance of the inactive wire.

The electrical power *P* drawn from the actuator is calculated as follows.

(2) P=U·I

In addition to the electrical validation, an infrared camera (ImageIR 8300, InfraTec, Dresden, Germany) was used to measure the temperature of the wire surface with the different electrical activation parameters. A telephoto lens with a focal length of 100 mm and a field of view of (9.1 × 7.3)° was employed to ensure optimal temperature detection.

## 3. Results and Discussion

### 3.1. Influence of Activation Parameters and Temperature on Deflection

**Figure 4:**
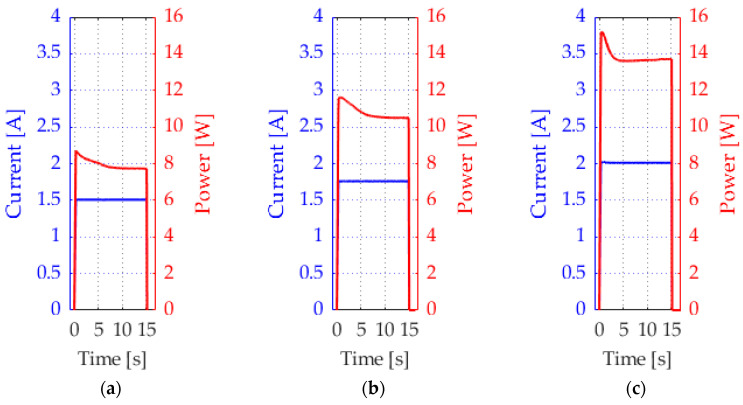
Electrical signals measured on wire surface, set with the parameters: (**a**) 1.5 A; (**b**) 1.75 A; (**c**) 2 A.

Figure 4 presents the measured current signals as well as the resulting electrical energy of selected activation sequences of the activations of 15 s. The set current was constantly achieved. The measured values match the values displayed on the power supply.

**Figure 5:**
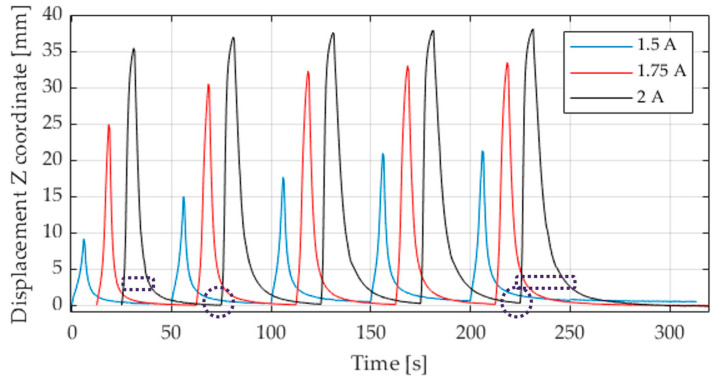
Displacement in Z direction for different actuation modes, 5 s actuation, 45 s switch-off time, time-shifted for better visibility.

An overview of some of the activation sequences is depicted in Figure 5. The most uniform deflection is achieved with the initially selected set-on time of 5 s and a set-off time of 45 s, with 2 A. At higher activation parameters, the deflection does not increase. When applying 1.75 A at the same times, the deflection is initially lower and then increases steadily over the recorded cycles. A further reduction in the current level reduces the deflection further. Regarding deflection, it should be noted that the actuators only reach their maximum deflection after several cycles of operation, as thermal equilibrium has not yet been reached at the beginning. The difference between the first activation cycles differs depending on the electrical power input. Furthermore, the measurements presented here demonstrate that, with increasing number of cycles, the actuator does not return completely to its initial state during the cooling phase. Additionally, it can be observed that the deflection peaks at the end become wider as the number of cycles increases, which means that the actuator slows down during the return movement.

**Figure 6:**
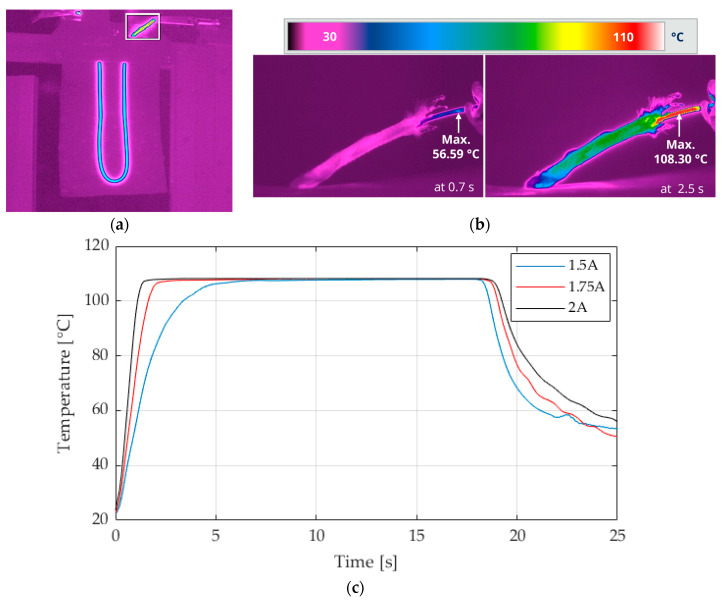
Measured temperature of wire surface. (**a**) Overview of the active actuator with highlighted wire measurement detail, shown in (**b**); (**b**) SMA wire at two temperatures during activation at the setting of 2 A 10 V; (**c**) temperature over 15 s at different electrical activation parameters.

Figure 6 displays the temperature of the SMA wire during activation with 2 V at two different times, as well as the temperature change in the wire with the extended switch-on time of 15 s of the same activation parameters, as shown in Figure 5. The results show that the actuator deflection is significantly influenced by the time, and the temperature of the wire increases. With increasing temperature, the wire contracts more, and the actuator has a higher deformation. Only slight differences in the maximum temperature can be observed on the surface of the activated wire. In general, the activation temperature of the wire is reached slightly faster with a higher current.

When a current of 1.5 A is applied, the behavior differs from that observed at higher current levels. The temperature rises more slowly at lower activation currents, and the actuation deflection is lower because of the Joule effect. The heat generation is non-linear due to the quadratic function.

(3) $$P=I^{2}\cdot R$$

Within the operational limits of the SMA, the saturation temperature of the SMA differs only slightly despite the steps in the current and thus the power (Figure 6). It is limited by the increasing resistance and heat dissipation, which increase at higher temperatures and are in equilibrium. The phase transition from martensite to austenite is complete, and the heat dissipation mechanism becomes more prominent. The current limitation restricts the heat generation and, thus, the wire temperature.

**Figure 7:**
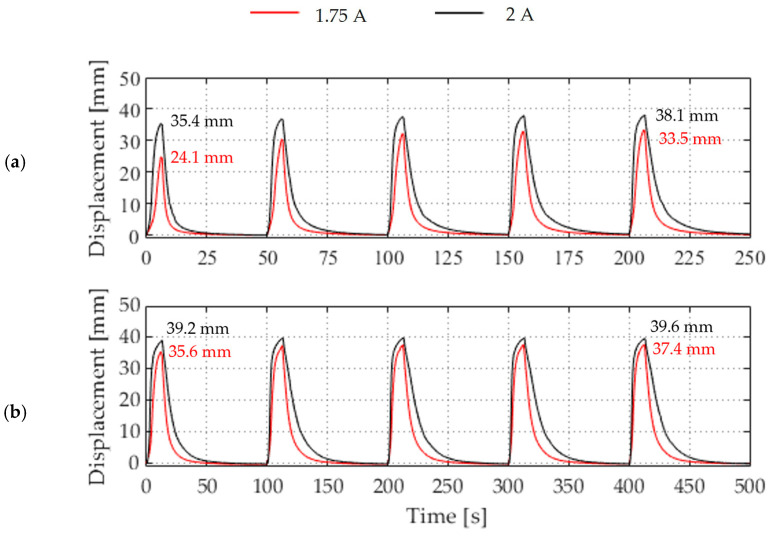
Displacement of the Z coordinate of the evaluated soft actuator configuration of switch-on and switch-off times at two different electrical activation modes and times: (**a**) 5 s switch-on and 45 s switch-off time, (**b**) 10 s activation and 90 s switch-off time.

Moreover, the two activation cycles with reduced current demonstrate a more pronounced increase in the deflection curve up to the maximum deflection, compared to 2 A. It can be observed that the deflection curve approaches a plateau towards the maximum, which indicates that the actuator is approaching its maximum deflection. This becomes evident when comparing 1.75 A and 2 A more precisely. At 1.75 A, the wire does not reach the same temperature within the switch-on time of 5 s, and the deflection is lower compared to 2A. However, by doubling the switch-on and switch-off times, the temperature can be reached, and the deflection is equalized. This is shown in Figure 7. Furthermore, it can be observed that the deflection is higher from the outset with increased times and increases less over the repetition cycles. This phenomenon is particularly evident when less current and power are achieved. However, even at 2 A, the deflection limit has still not been reached at 5 s, as evidenced by the generally higher and more equal deflection at the switch-on time of 10 s. The maximum deflection of the actuator was reached during activation with a current flow of 2 A.

When precise timing and minor deflection losses are not crucial, a lower current flow with a longer activation time can be chosen for activation. The actuator’s deflection is less for the same switch-on time at a lower current. Small electrical fluctuations have no noticeable effect.

**Figure 8:**
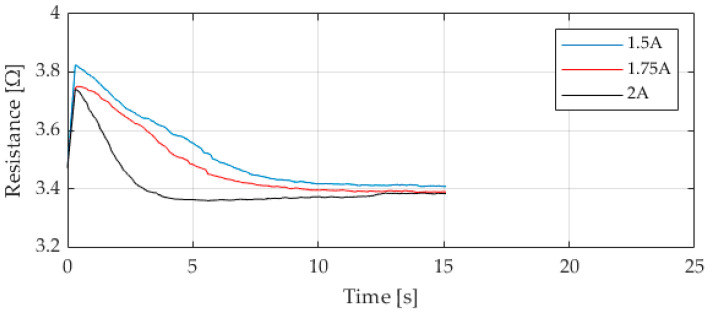
Calculated resistance at different electrical activation parameters over 15 s.

The resistance curves shown in Figure 8, calculated from the measured electrical signals, follow the known behavior of SMA materials and show the phase change rate. After switching on the power source, the resistance is initially high. As the temperature increases, the resistance decreases during the phase transition and remains constant at the same temperature after the transformation. As expected, the resistance is similar to the temperature curve, which matches the current limitation. At higher currents, it falls faster, and at lower currents, it takes longer to stabilize, as expected.

### 3.2. Three-Dimensional Actuator Deflection

**Figure 9:**
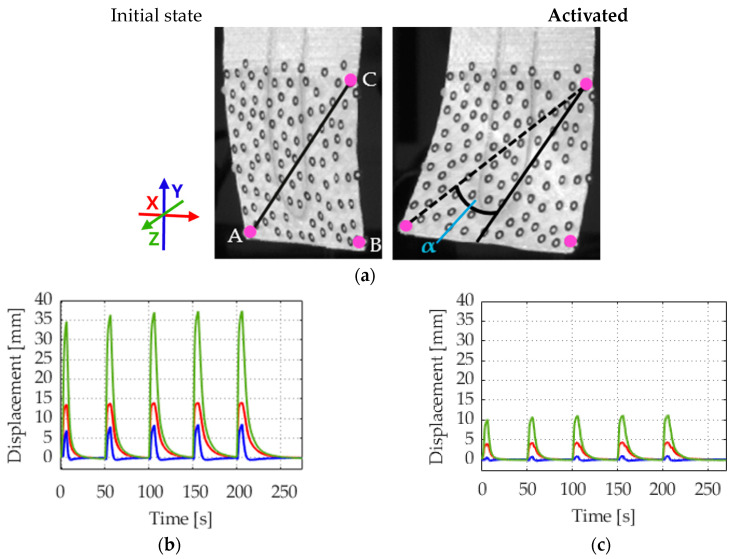

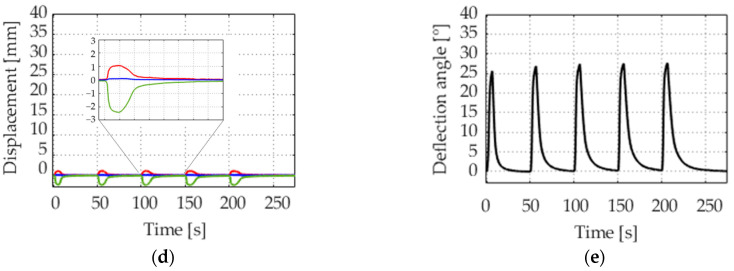
Three-dimensional deflection of an actuator, activated with 2 A. (**a**) Overview of the actuator with characterized details; (**b**) displacement point A: left down; (**c**) displacement point B: right down; (**d**) displacement point C: transition between the textile layers; (**e**) deflection angle α.

To characterize the three-dimensional deformation behavior, the graphs of the deflection of three characteristic points on the surface of an actuator are presented in Figure 9. The characterization is employed to evaluate the torsional and bending deformation of the current actuator and to evaluate and improve the potential performance and limit of the overall three-dimensionality of future actuators. An overview of the position on the surface of the points is provided in Figure 9a. The characterization of points A to C and the angle constructed from them was carried out from one measurement.

The actuator shows, as expected, torsional and bending actuation. Point A (Figure 9b) shows the most pronounced deformation. The highest displacement is in the Z direction. A comparison of the displacement in the X and Y directions reveals a notable difference, with a higher displacement in the X direction. This indicates the presence of torsional movement, which is influenced by the mechanical anisotropy introduced by the overstitched layer of fabric. Point B (Figure 9c) on the other edge also shows deflection, but less than A.

Point C, shown in Figure 9d, is on the borderline of the two distinct textile reinforcements, as illustrated in Figure 1. It demonstrates a minor displacement in the Z direction, in the opposite direction to the other two points. The same can be observed on the opposite side at the other vertical edge, which is not explicitly indicated.

The angle α (Figure 9e) is maximum up to 28°. When considered in conjunction with the aforementioned information from the analyzed points, it can be concluded that the actuator exhibits a greater tendency to bend than to twist when activated. The Z direction is equated with bending, and the *X*-axis with twisting.

### 3.3. Maximum Actuation Due to Overheating of the Actuator

The soft actuator demonstrated the capacity to be activated for up to 15 s without encountering any issues when activated with 2 A. However, the deflection no longer increased over the switch-on time towards the end. In addition to the activation by the SMA wire, the limitation due to the textile structure of the actuator is a significant factor in this regard. The degrees of freedom resulting from the anisotropic orientation of the corresponding textile layer, as illustrated in Figure 1, establish the upper limit for the maximum attainable deflection. When activated with a 5 s switch-on time and 35 s switch-off time, the composite structure had still not failed after 500 activations. Consequently, the total activation time was 60 min. Nevertheless, a reduction in deflection of around 30% was already observed after this time. To date, delamination of the structure has only occurred in instances where the structure was already damaged during the manufacturing process.

**Figure 10:**
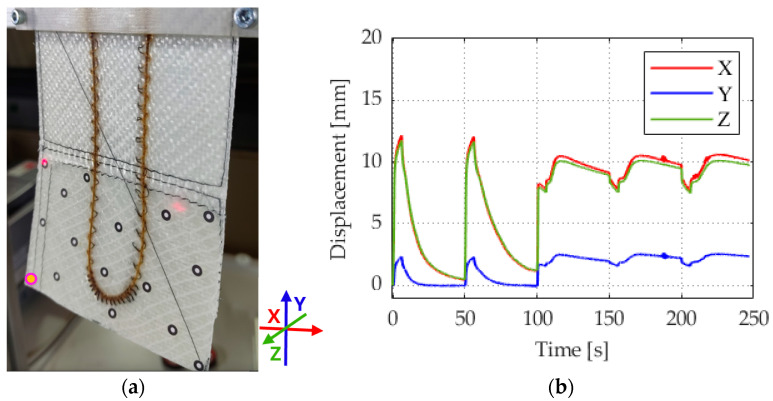
(**a**) Image of a damaged actuator and (**b**) deflection curve of reduced actuation and fatigue of an actuator, activation with activation setting of 3A, 10 V.

The sample became visibly overheated with prolonged activation, and severe damage was expected. The power supply was therefore switched off. The SMA wire was quickly damaged at higher power levels than those specified in Section 3.1. When activated with 3 A, this occurred during the recording of the deflection. Figure 10b shows a case of successive overheating leading to reduced actuation and failure of the actuator. Due to the overheating, the polyamide threads of the braiding of the SMA wire turned brown. This can be observed by comparing Figure 1a and Figure 10a. This phenomenon can be attributed to the onset of oxidation and the decomposition of additives in the material. The initial deflection of the actuator was comparable to point A in Figure 9 but with more torsional deflection.

Overheating changes the material’s phase structure and the SMA wire’s ability to retain its shape. The wire cannot contract to the same extent as it would have if undamaged.

## 4. Conclusions

In this work, a multi-sensor camera system with four camera pairs is used to analyze the 3D deflection of a fiber-reinforced soft actuator driven by an SMA wire. Five activation sequences of the actuator were recorded and analyzed using DIC. In addition, an IR camera was implemented in the test stand to monitor the temperature of the actuated actuator of the wire, which enabled a more precise comparison of the wire temperature with the activation parameters. The analysis method was employed to examine the impact of the activation parameters, namely activation voltage, activation time, and repeated activations, on the torsional deflection. The test setup was optimally configured to investigate the deflection of the actuator. It is particularly important that, with optimal positioning of the cameras, the video sequences captured by the camera system can be analyzed to identify several points of interest on the test object using a single measurement. Thus, the 3D analysis of the structures enables the use of increasingly complex structures in future studies.

If an activation time of 5 s is selected, several activations, e.g., cyclic movement, are necessary until a uniform deflection is achieved. However, if the activation time is increased to 10 s, a higher deflection is achieved with a one-time switch-on and the same electrical parameters. The optimal deflection has thus far been achieved with a current flow of 2 A.

The data acquired regarding the power settings and deflection of the actuator with an anisotropic structure provide information to further develop the control and regulation for similar structures.

## Data Availability

Data are contained within the article. The raw data supporting the conclusions of this article will be made available by the authors on request.
